# An efficient DNAzyme for the fluorescence detection of *Vibrio cholerae*

**DOI:** 10.1002/fsn3.3304

**Published:** 2023-03-13

**Authors:** Qingzhen Miao, Wen Ding, Xiuli Bao, Siyuan Wang, Qianru Lin, Yingying Xu, Jing Lu, Mingsheng Lyu, Shujun Wang

**Affiliations:** ^1^ Jiangsu Key Laboratory of Marine Bioresources and Environment/Jiangsu Key Laboratory of Marine Biotechnology Jiangsu Ocean University Lianyungang China; ^2^ Co‐Innovation Center of Jiangsu Marine Bio‐industry Technology Jiangsu Ocean University Lianyungang China

**Keywords:** DNAzyme, fluorescence detection, onsite detection, sensitive *V. cholerae* biosensor

## Abstract

*Vibrio cholerae* (Vc) causes cholera disease. Vc contamination is widely found in water and aquatic products, and therefore is a serious food safety concern, especially for the seafood industry. In this paper, we attempted the rapid detection of *V. cholerae*. Nine rounds of in vitro selection using an unmodified DNA library were successfully performed to find specific DNAzymes of Vc. Their activity was evaluated based on a fluorescence assay and gel electrophoresis. Finally, a DNAzyme (named DVc1) with good activity and specificity with a detection limit of 7.2 × 10^3^ CFU/mL of Vc was selected. A simple biosensor was constructed by immobilizing DVc1 and its substrate in shallow circular wells of a 96‐well plate using pullulan polysaccharide and trehalose. When the crude extracellular mixture of Vc was added to the detection wells, the fluorescent signal was observed within 20 min. The sensor effectively detected Vc in aquatic products indicating its simplicity and efficiency. This sensitive DNAzyme sensor can be a rapid onsite Vc detection tool.

## Graphical abstract

Nine rounds of in vitro selection using an unmodified DNA library were successfully performed to find specific DNAzymes of Vibrio cholerae.A DNAzyme (named DVc1) with good activity and specificity with a detection limit of 7.2×10^3^ CFU/mL of Vc was selected. A simple biosensor based on DVc1 can effectively and fast detected *V. cholerae* in aquatic products.

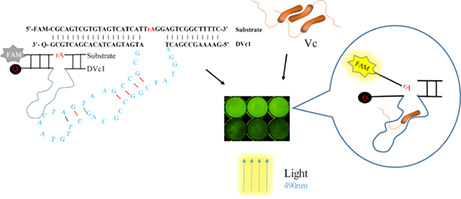

## 1 INTRODUCTION

Cholera, a virulent infectious disease with frequent outbreaks and high mortality rates, affects millions of people causing tens of thousands of deaths each year (Chowdhury et al., 2021). It is classified as one of the international quarantine infectious diseases (Balasubramanian et al., 2021). *Vibrio cholerae* (Vc), a gram‐negative bacteria of the *Vibrio* genus, causes cholera. Having poor sanitation and hygiene problems, many developing countries had frequent outbreaks of cholera. So far, more than 200 types of Vc strains have been identified based on the variation of the “O” antigen (Banerjee et al., 2014; Das et al., 2016); the strains belonging to serogroups O1 and O139 are associated with epidemic cholera. Vc infection spread through food or water contamination. Most of the ingested Vc bacteria are killed by gastric acid, however, those that survive cause infection through two main virulence factors: toxin‐coregulated pilus (TCP) and cholera toxin (CT). TCP helps Vc to colonize the mucosal layer of the host's intestine, where the pathogen releases CT that crosses the gastro‐endothelial wall causing acute watery diarrheal disease in humans (Hun Yoon & Waters, 2019). One of the current Vc detection methods is cell culture, which takes longer and therefore is not suitable for processing multiple samples and lacks rapid detection. Immunology‐based Vc detection methods include ELISA (Bayat et al., 2018), immunofluorescence techniques (Wang et al., 2010), colloidal gold detection techniques (Peng & Chen, 2018), and speckle hybridization techniques (Pengsuk et al., 2011), however, these methods are cumbersome and have high false positives and low sensitivity. Novel Vc detection methods include nucleic acid‐based polymerase chain reaction (PCR) (Guan et al., 2021) and isothermal amplification techniques (Zhang et al., 2015), which require equipment and highly trained personnel. Vc infections are pretty common in aquatic products and there have been increasing reports about Vc contamination of raw seafood. This is a serious food safety problem, especially for the seafood industry. Early detection of Vc contamination can help disease prevention and therefore demands a simple and rapid detection method.

Functional nucleic acids (FNA) are nucleic acids and nucleic acid mimetic molecules (such as DNAzymes (Douglas et al., 2009; Joyce, 2001; Malyshev et al., 2014), nucleic acid aptamers (Hamilton & Baulcombe, 1999), DNA tiles, DNA origami (Douglas et al., 2009), and other types of nontraditional nucleic acids (Ambros et al., 2003; Hamilton & Baulcombe, 1999; Malyshev et al., 2014)) that can replace traditional proteases and antibodies and have molecular recognition capabilities. A DNAzyme with biocatalytic activity can perform specific biological nongenetic functions. The first RNA‐cleaving DNAzyme was reported in 1994 (Breaker & Joyce, 1994), and since then many different DNAzymes have been screened (Silverman, 2005, 2016). DNAzymes are short single‐stranded catalytic DNA molecules with substrate recognition capability. Although no natural DNAzymes have been identified so far, the development of the Systematic Evolution of Ligands by Exponential Enrichment (SELEX) technique allows isolating specific DNAzymes from a synthetic library of approximately 10^14^ random DNA sequences (Breaker & Joyce, 1994). RNA‐cleaving DNAzymes can be attached with a fluorescent reporter group (named RNA‐cleaving fluorogenic DNAzyme, RFD) to generate a fluorescent signal upon binding to an appropriate target. RFDs are designed to perform three sequential functions: recognition of bacterial markers, cleavage of RNA, and finally the generation of a fluorescence signal. RFDs can be used to set up a simple “mix‐and‐read” bacterium detection assay, where a fluorescence signal confirms the presence of the target bacteria (Zhang et al., 2016). In this work, the catalytic structural domain of the RFD consists of 35 deoxyribonucleotides flanked by two substrate recognition structural domains, 20 and 12 bases on the respective side (Santoro & Joyce, 1998). The substrate cleavage site containing a ribonucleotide (rA) is embedded in the RFD DNA sequence. Also, the RFD has a fluorophore (F) at the 5′ substrate binding end and a quencher (Q) at the 3′ end of the DNAzyme. Fluorescence is quenched in absence of the target or at very low concentration, however, once the target binds to the DNAzyme with the assistance of metal ions, the substrate is cleaved releasing the quenching group thus generating a fluorescent signal (Zhang et al., 2016).

Several DNAzymes have been screened for high affinity, selective binding to specific target molecules, and additional catalytic functions for in vitro applications. The DNAzyme catalytic function can convert molecular interactions into visible signals (luminescence or color change). The size of a DNAzyme can be from 20 to over 100 nucleotides. DNAzymes are chemically and thermally stable and can be easily synthesized. Also, the internal bases or terminals of a DNAzyme can be modified to provide additional functionality. DNAzymes have been used for the detection of metal ions (such as Ni^2+^ (Ren et al., 2020), Zn^2+^ (Huang et al., 2020), Pb^2+^ (Fu et al., 2016), K^+^ (Fan et al., 2012), and Na^+^ (Sun et al., 2016)), bacteria (Ali et al., 2011; Ali, Slepenkin, et al., 2019; Ali, Wolfe, et al., 2019; Gu et al., 2019; Shen et al., 2016), toxic algae (Bernardinelli et al., 2020), tumors (Xue et al., 2019), histidine (He et al., 2015), insulin (Ma et al., 2018), ascorbic acid (Malashikhina & Pavlov, 2012; Miao et al., 2012), glucose (Liu et al., 2015; Yang et al., 2015), and thrombin (Sun et al., 2018). DNAzymes combined with nanomaterials have been used for targeted drug delivery (Marquardt et al., 2015) for the treatment of cancer (Eicher et al., 2019) and other diseases (Yang et al., 2021), and many biosensors and biomedicine applications.

RFDs have been used for the detection of some pathogenic bacteria such as *Escherichia. coli* (Ali et al., 2011), *Clostridium difficile* (Shen et al., 2016), *Vibrio anguillarum* (Gu et al., 2019), *Klebsiella pneumoniae* (Ali, Slepenkin, et al., 2019), *Helicobacter pylori* (Ali, Wolfe, et al., 2019), *Aeromonas hydrophila* (Ma et al., 2021), *Pseudomonas aeruginosa* (Qin et al., 2021), *Vibrio vulnificus* (Fan et al., 2021), and *Legionella pneumophila* (Chang et al., 2020, 2021; Rothenbroker et al., 2021). Here, we screened a specific DNAzyme against Vc and studied its characteristics to successfully construct a DNAzyme‐based biosensor for the rapid detection of Vc. To our knowledge, this is the first DNAzyme‐based Vc detection method that can help the management of Vc infection/contamination.

## 2 MATERIALS AND METHODS

### 2.1 Preparation of the crude extracellular mixture of Vc

Glycerol‐preserved Vc was inoculated in 20 mL of Vc exclusive medium that contained 3.5% sodium chloride agar medium (beef dip (1:3) 1.0 L, peptone 10.0 g, NaCl 35.0 g, agar 20.0 g, pH 7.2 ~ 7.4). The culture was incubated at 25°C and 180 RPM for 15–20 h until the OD_600_ (optical density at 600 nm) of 1. A part of the cultured bacterial broth was transferred to a 1.5‐mL sterilized EP tube and centrifuged at 5000 RPM for 5 min. The obtained supernatant is the crude extracellular mixture of Vc (CEM‐Vc), which was stored at −20°C for subsequent experiments; the precipitate was discarded. The rest of the bacterial culture was diluted in a gradient manner; 100 μL of each dilution was spread on 3.5% NaCl agar medium and the colonies were counted after incubation for 36 h at 25°C.

### 2.2 Preparation of the crude extracellular mixture of other bacteria

*Pseudomonas aeruginosa* (Pa), *Vibrio shilonii* (Vs), *Vibrio harveyi* (Vh), *Escherichia coli* (Ec), *Staphylococcus aureus* (Sa), *Bacillus subtilis* (Bs), and *Vibrio anguillarum* (Va) were cultured in LB media (Luria–Bertani Broth, 1% tryptone, 0.5% yeast extract powder, 1% NaCl, pH 7.0) at 30°C and 180 RPM for 12 h up to the OD_600_ of ~1. The crude extracellular mixtures were prepared as described for Vc in section 2.1 and stored at −20°C. All strains were purchased from China Industrial Microbial Culture Collection Management Center (Beijing, China).

### 2.3 In vitro selection

In this study, we used the SELEX technique for in vitro selection. The forward and reverse primers and the ends of the random DNA library were designed in‐house, synthesized by Sangon Biotech (Shanghai) Co., Ltd, and purified by 10% denaturing polyacrylamide gel electrophoresis (dPAGE). The relevant oligonucleotide designs are shown in Table 1. The oligonucleotide sequence of the forward primer contains a biotin tag and adenine oligonucleotide (named rA), which were ligated into the library by PCR. The forward primer and rA serve as the substrate for the DNA library and cleavage junction, respectively.

**TABLE 1:** DNA sequences used in this study.

Name	Oligonucleotide sequences (5′ ~ 3′)
Lib	Phosp‐GGAGTCGGCTTTTCCCGACT‐N35‐ATGATGACTACACGACTGCG
FP	Biotin‐TAGTCATCATTrAGGAGTCGGCTTTTC
RP	CGCAGTCGTGTAGTCATCAT
DVc1	GAAAAGCCGACTAGGTATCGGCCGCAGCTTGTAACTAGTAAGCCGCG ATGATGACTACACGACTGCG‐Q
Substrate	FAM‐CGCAGTCGTGTAGTCATCATTrAGGAGTCGGCTTTTC

*Note*: The library and primer sequences are listed in Table 1.

*Abbreviations*: Lib, library; FP, forward primer; RP, reward primer; Phosp, phosphorylation; N35, 35 random nucleotides; rA, adenosine ribonucleotide; Q, quencher; FAM, fluorophore.

**FIGURE 1:**
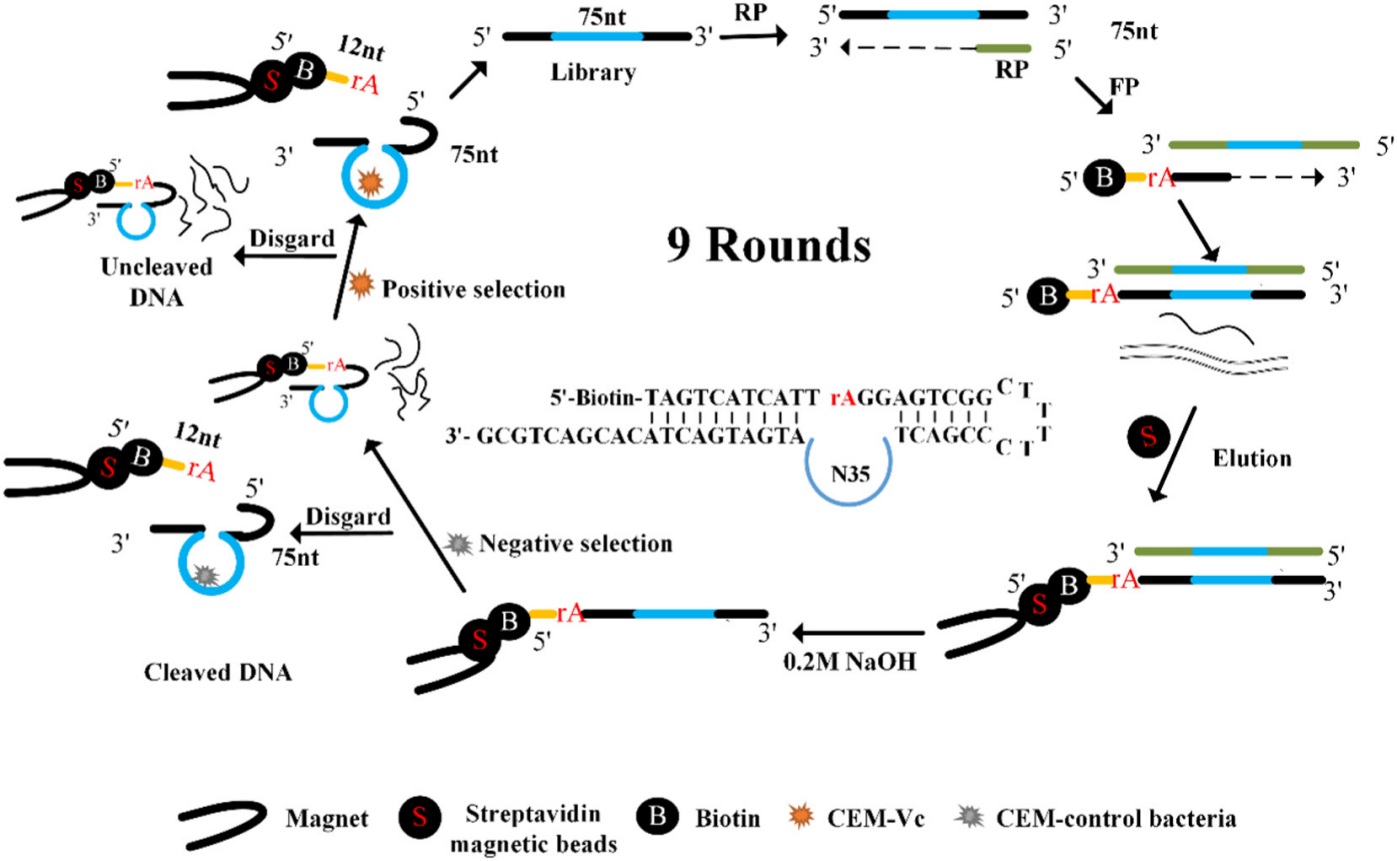
Scheme of the DNAzyme selection. There are 35 nt random nucleotide in the library, and nine rounds of selection was performed. Positive selection was performed in the 1st, 3rd, 5th, 7th, and 9th rounds of the selection process. Negative selection was performed only in the other rounds. The cleavage site is at the rAG junction and biotin was labeled on the 5′ end. The target molecule is crude extracellular mixture (CEM).

The DNA library (Lib) contains a 35‐nt‐long random sequence domain (called N35), forward primer (FP), and reverse primer (RP) purified by 10% dPAGE. To begin the selection process, PCR was conducted to attach the substrate with the DNA library (10–100 ng/μL); each PCR contained equal amounts of FP and RP (10 μM each), 10× PCR buffer (100 mM Tris–HCl, pH 8.8 and 500 mM KCl, 0.8% (v/v) NP‐40), 25 mM MgCl_2_, dNTP mixture (including dATP, dCTP, dGTP, and dTTP, 10 mM each), Taq DNA polymerase (5 U/μL), and ultrapure water. The PCR was performed as follows: predenaturation at 94°C for 5 min, denaturation at 94°C for 30 s, annealing at 55°C for 30 s, extension at 72°C for 1 min, and final extension at 72°C for 3 min. The reaction was cooled and stored at 4°C. A total of 27 PCR cycles were performed, and the PCR products were sequentially retrieved at cycle numbers 9, 11, 13, 15, 17, 19, 21, 23, 25, and 27 to find the optimal number of cycles by 2% agarose gel electrophoresis. The final PCR enrichment reaction was performed according to the optimal number of cycles. The selection process is shown in Figure 1: (1) The DNA library after PCR contained biotin that can be attached to streptavidin‐coated magnetic beads. (2) The DNA library was counter‐screened by incubating with CEM‐control bacteria (Pa, Vs, Vh, Ec, Sa, Bs, Va); cleaved DNA was discarded, and the uncleaved DNA was collected for the next round of positive selection. (3) The DNA, which was bound to CEM‐Vc with a specific structure and can perform the cleavage reaction, was used for the next round of selection. (4) Active sequences were efficiently added using PCR and enriched in the next round of selection. (5) The total selection process included nine rounds. The positive selection was performed in the 1^st^, 3^rd^, 5^th^, 7^th^, and 9^th^ rounds of the selection process. The negative selection was only performed in the other rounds. After the ninth round of enrichment, the PCR products were recovered by alcohol precipitation and dried, which were later dissolved in 100 μL of ultrapure water and sent to Sangon Biotech for high‐throughput sequencing.

### 2.4 Screening of active DNAzyme

The five highly enriched sequences obtained from high‐throughput sequencing were used as candidate sequences. The candidate DNAzymes and substrate were synthesized by Sangon Biotech; each candidate DNAzyme was individually ligated to its substrate to examine activity. The DNAzyme–substrate complex (named DVc‐S) was prepared by a reaction mixture containing 2.5 μM substrate, 2.5 μM DNAzyme, and 2× Selection buffer (SB; 100 mM HEPES, pH 7.5, 300 mM NaCl, 30 mM MgCl_2_ and 0.02% Tween 20). The reaction was conducted in a boiling water bath for 3 min and cooled naturally at room temperature (RT). The assay reactions were divided into experimental (with CEM‐Vc) and control (without CEM‐Vc, replaced with exclusive medium) groups. 4 μL DVc‐S (Q at the 3′ end of the substrate strand S and fluorescent FAM label at the 5′ end of DNAzyme strand DVc), 41 μL of ddH_2_O, 2× SB (2 × Selection buffer), and 45 μL were mixed well and added to a well of a 96‐well plate. The fluorescence signal was measured by a microplate reader (excitation wavelength, 485 nm; emission wavelength, 535 nm) as the initial fluorescence value F0. Next, 10 μL of the exclusive medium (control reaction) or CEM‐Vc (experimental reaction) was added to the respective wells and the reactions were performed for 2 h. The fluorescence value was measured as F. The reactions had three parallel replicates. The lysis activity is expressed as F‐F_0_. The fluorescence was detected at 30‐s intervals (Infinite M1000Pro, Tecan).

Also, to examine the cleavage reaction, reaction mixtures were analyzed by dPAGE. For the fluorescence assay, the reaction mixtures were transferred to light‐proof tubes, and the reactions were terminated by adding 2 × gel loading dye blue (containing 8 M urea) (2:1). Also, the DNA Marker (2:1) was added to the termination solution as a control.

Each well was rinsed with 1 × TBE to flush out the precipitated urea. 15 μL of the sample mixture and Marker were resolved by gel electrophoresis at 120 V for 50 min. The resolved gel was imaged and quantitatively analyzed (cleaved DNA + uncleaved DNA = 100%) by a Bio‐Rad GelDoc™ EZ imaging system (BIO‐RAD).

### 2.5 Specificity of DVc1

The control bacteria (Pa, Vs, Vh, Ec, Sa, Bs, Va) were cultured in LB media at 30°C and 180 RPM for 12 h up to OD_600_ = 1. The bacterial broths were centrifuged at 5000 RPM for 5 min. The obtained supernatants were used as respective CEM, which were tested in fluorescence and cleavage assays as described above for CEM‐Vc.

### 2.6 Optimization of reaction conditions

#### 2.6.1 pH

The optimized pH range of the biosensor assay was 4.0–9.5; 100 mM HEPES (4‐(2‐hydroxydiformyl) piperazine‐1‐dimethylsulfonic acid) was used as a buffer with 300 mM NaCl, 30 mM MgCl_2_ and 0.02% Tween‐20. Accordingly, 2× SB buffer pH was adjusted with HCl and NaOH. DVc1 was reacted with CEM‐Vc at different pH and the results were compared based on the intensity of the fluorescence signal.

#### 2.6.2 Metal ions

The optimal concentrations of Na^+^ and Mg^2+^ in the used buffers were optimized at pH 8. In the absence of Mg^2+^, different amounts of Na^+^ (0, 30, 60, 90, 120, 150, 180, 210, 240, 300, 400, and 600 mM) were tested to select the optimal Na^+^ concentration for the highest cleavage activity of DVc1. Then, different amounts of Mg^2+^ (0, 30, 60, 90, 120, 150, 180, 210, 240, 300, 400, and 600 mM) at optimal Na^+^ concentration were tested to find the optimal Mg^2+^ concentration. Ethylene Diamine Tetraacetic Acid (EDTA) was added to 2× selection buffer to determine the effect of no metal ions. Likewise, the effect of different divalent metal ions (Co^2+^, Zn^2+^, Mg^2+^, Ba^2+^, Mn^2+^, Ca^2+^, Sr^2+^, Cu^2+^, Fe^2+^) on the cleavage activity of DVc1 was tested at the optimal concentration of Na^+^.

### 2.7 Sensitivity detection of DVc1

The initial Vc culture was gradually diluted with 2× selection buffer in a 10‐fold gradient (10^1^–10^8^), and the diluents were added to the DVc1‐S mixture to determine the sensitivity range of the DNAzyme by the fluorescence and gel assays. 200 μL of initial Vc culture and 1.8 mL of 2× selection buffer were added to 2 mL EP tubes for 10‐fold gradient dilution (10^1^–10^8^). Then, 4 μL of DVc‐S (2.5 μM substrate 2.5 μM DVc1), 41 μL of ddH_2_O, 45 μL of 2 × SB (2 × Selection buffer), and 10 μL of the diluents were added to the wells of a 96‐well plate. The total reaction volume was 100 μL. The reactions were performed for 2 h, the fluorescence signal was measured, and then the sample solution was taken for dPAGE analysis.

### 2.8 Properties and molecular weight of the target

It is difficult to find an effective target molecule in CEM, which is a complex mixture of proteins. Several studies suggested that DNAzyme targets are mostly proteins (Ali, Slepenkin, et al., 2019; Ali, Wolfe, et al., 2019; Shen et al., 2016). Accordingly, we assumed that there is a target protein in CEM‐Vc. So, we used Proteinase K to digest (37°C for 1 h) 30 μL of CEM‐Vc, which was subjected to fluorescence detection for 2 h. The CEM‐Vc and Blank groups had CEM‐Vc and medium, respectively. Meanwhile, both the whole cell and cell lysate were detected. The bacterial broth was centrifuged at 5000 RPM for 5 min to remove the CEM of Vc, and then, the precipitant was mixed with medium to recover the volume of the whole cell precipitate. The cell lysate was obtained by sonication of the whole cell. The medium was used as the blank group.

The molecular weight of the identified target was evaluated using different pore sizes (10, 30, 50 and 100 kDa) of ultrafiltration membranes. CEM‐Vc was ultrafiltered and then the corresponding lower filtrates were tested by cleavage and fluorescence assays.

### 2.9 Biosensor board design

The polystyrene plate cover of a 96‐well plate with 96 shallow circles was used as the sensor platform, where the DNAzyme was immobilized. Briefly, DVc1‐S was mixed with 2× SB, 8% pullulan polysaccharide, and 0.25 M alginose (prepared to keep away from light) in a light‐proof tube. Of this, 30 μL was applied to the shallow circles, the plate cover was covered with tin foil, and then the plate was incubated at 60°C for 1 h. Next, 25 μL of CEM‐Vc (experimental group) or medium (blank group) was added to the shallow wells for 2 h, and the fluorescence was measured with the naked eye under a LED transilluminator.

Also, the DVc1‐S concentration (0.02, 0.04, 0.06, 0.08, 0.1, 0.14, 0.2, and 0.3 μM) and CEM‐Vc reaction time (within 1 h) were optimized. The sensor sensitivity was tested as described in section 2.7. The sensor specificity was measured against the CEM control bacteria (section 2.5).

### 2.10 Detection using the sensor board

Raw choking sea crab forceps, raw choking oysters, and cold jellyfish were obtained from the local market and thoroughly rinsed with tap and then pure water. The above four products were divided into two groups of equal amounts (10 g each) as the experimental and control groups, which were added with 4 mL of bacterial solution or ultrapure water, respectively. After mixing, 25 μL of the test sample was added to the sensor plate for 20 min and then fluorescence was measured and photos were obtained. Also, the experimental group was diluted 10 times for the detection limit test. The blank group was diluted with ultrapure water.

### 2.11 Data analysis

All the experiments had been set as three parallel samples. And the data were analyzed by SPSS v20. The bar or dot in the figures stood for mean ± SD. Significant (*p* < .05) was marked as a different letter.

## 3 RESULTS

### 3.1 Screening of active DNAzyme

**FIGURE 2:**
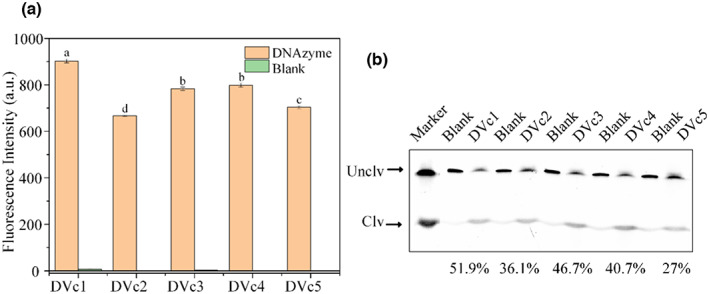
Activity assessment of the DNAzyme candidates. (a) Fluorescence intensity and (b) Gel‐based cleavage activity assessment of DVc1‐DVc5 DNAzymes [a‐d were considered to be statistically significant (*p* < .05), and the same letters were considered to be not significant (*p* > .05)]. The bar means: mean ± SD in the legend. D1‐D5:DVc1‐DVc5; Unclv: Uncleaved intact full‐length DNAzyme; Clv: cleaved product of the DNAzyme after the reaction. The marker lanes are completely cleaved DNA.

The highly enriched sequences obtained by high‐throughput sequencing are listed in Table 2 and the five sequences were selected as the candidates (Table 2). Fluorescence and cleavage (gel electrophoresis) assays were performed to compare the activities of the synthesized DNAzymes (Figure 2). Although all the DNAzymes showed cleavage responses to CEM‐Vc, DVc1 performed the best in both the fluorescence (highest intensity) and cleavage (higher cleavage) assays. Therefore, DVc1 was chosen for the subsequent experiments. The complete sequence of DVc1 and its substrate sequence are shown in Table 1.

**TABLE 2:** The results of high‐throughput sequencing and selected candidate DNAzymes.

Sequences of random region (N35, 5′—3′)	Length	Sequence number	Percentage	Name
AGGTATCGGCCGCAGCTTGTAACTAGTAAGCCGCG	35	10,362	20.13	DVc1
ATCAGTCATGAGCGTGGCAGTAGCGGTGCCGACGC	35	4245	8.25	DVc2
AAGGGGCGTAGCAGAAACAGTCGACGGTTCAACGT	35	3956	7.69	DVc3
GGGTCGACGCAGAAAATCGCCGAGCTCCGAAGATA	35	3387	6.58	DVc4
ACGGAGGTACGGAGGGAAAATCGTATGCAGGTCGC	35	1788	3.47	DVc5

### 3.2 Specificity of DVc1

**FIGURE 3:**
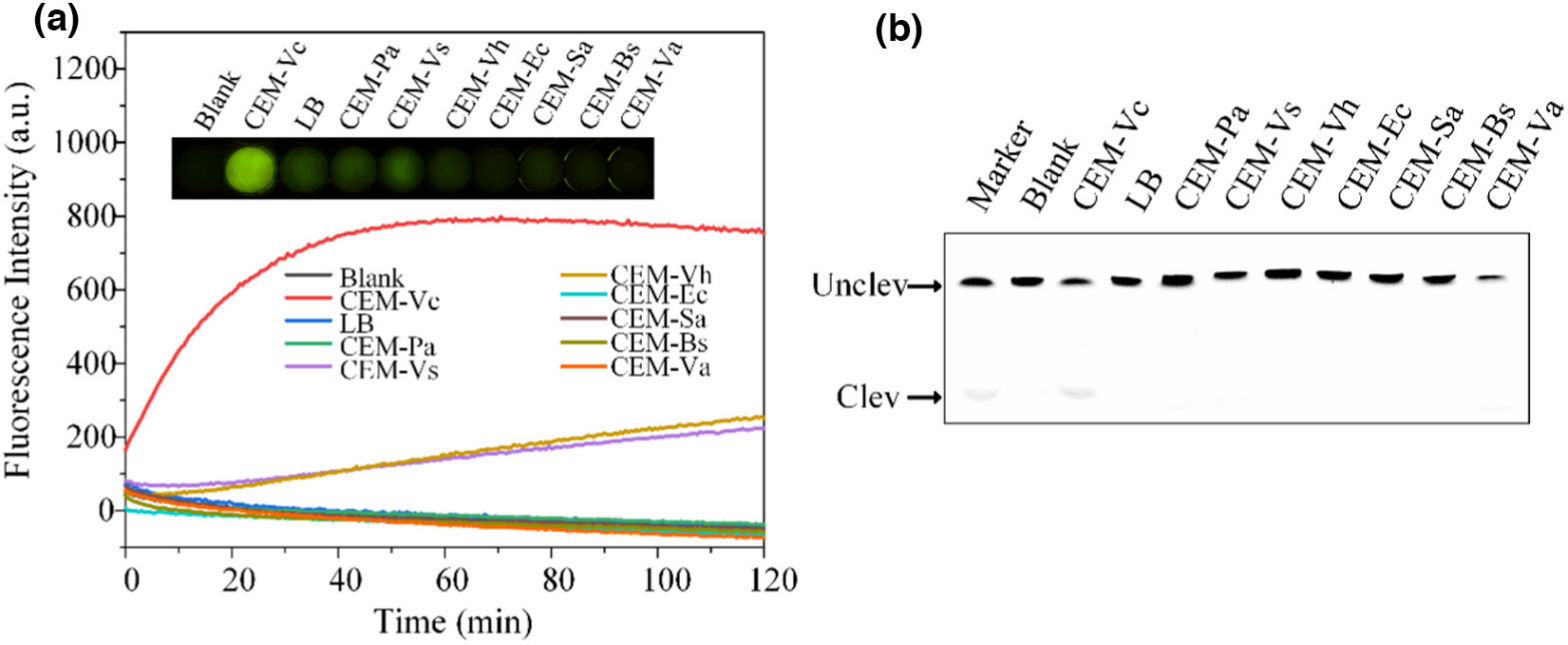
DNAzyme activated by CEM‐Vc. (a) DVc1 was tested with different bacterial CEM. Fluorescence biosensor‐based activity assessment of DVc1 against different bacterial CEM. The corresponding pictures is insert. (b) Gel‐based (15% dPAGE with 8 M urea) cleavage activity assessment of DVc1 against different bacterial CEM. *Pseudomonas aeruginosa* (Pa), *Vibrio shilonii* (Vs), *Vibrio harveyi* (Vh), *Escherichia coli* (Ec), *Staphylococcus aureus* (Sa), *Bacillus subtilis* (Bs), and *Vibrio anguillarum* (Va).

Fluorescence and cleavage analyses were performed to assess the selectivity of DVc1 (Figure 3a and b). As shown in Figure 3a, CEM‐Vc generated the highest fluorescence signal. The fluorescence intensity of Vc compared with one of the nonspecific signals was over seven times within 20 min. Also, only the CEM‐Vc could cleave DVc1 specifically (Figure 3b).

### 3.3 Optimization of reaction conditions

**FIGURE 4:**
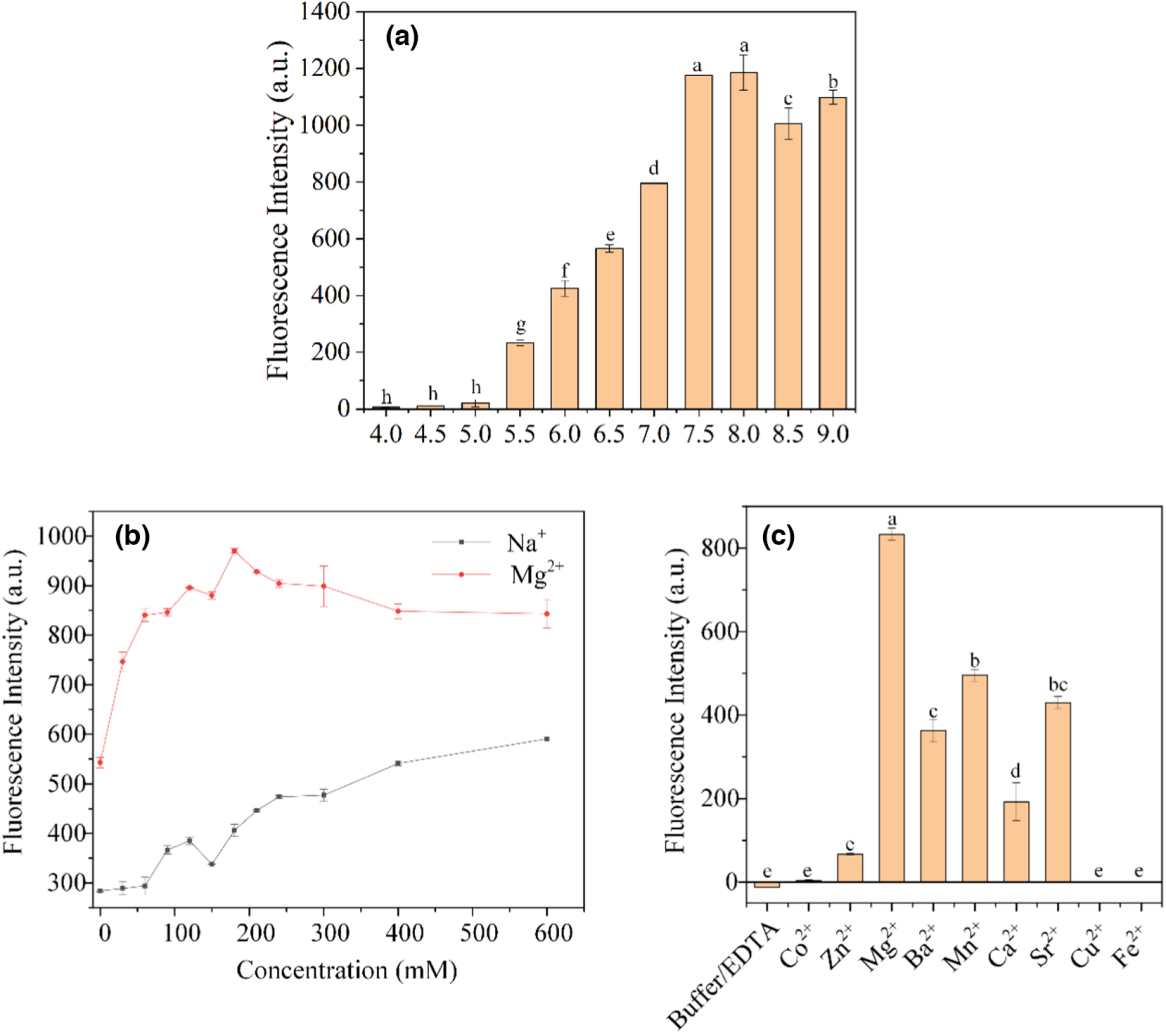
Optimization of experimental conditions. (a) pH optimization. Cleavage was performed with CEM‐Vc [a‐h were considered to be statistically significant (*p* < .05), and the same letters were considered to be not significant (*p* > .05)]. (b) Effects of Na^+^ and Mg^2+^ concentration and (c) various divalent metal ions on the cleavage activity of DVc1 [a‐e were considered to be statistically significant (*p* < .05), and the same letters were considered to be not significant (*p* > .05)]. The Buffer/EDTA reaction contained 300 mM EDTA in 2 × Selection buffer. The bar and the dot mean: mean ± SD in the legend.

The optimal reaction pH 8.0 was found as described in section 2.6.1 (Figure 4A). The fluorescence was abnormally high at pH 9.5, presumably caused by the high concentration of Na^+^. All subsequent experiments were carried out at pH 8.0. The results of metal ion concentration optimization are shown in Figure 4B. The cleavage activity of DVc1 increased with the increase in Na^+^; 300 mM Na^+^ was selected as the optimal amount. Meanwhile, DVc1 activity first increased between 0 and 180 mM Mg^2+^ and then decreased at higher Mg^2+^ concentrations, which could have altered the 3D structure of DNAzyme. Hence, 180 mM Mg^2+^ was chosen as the optimal amount. Moreover, DVc‐1 showed no cleavage activity in the absence of divalent metal ions (Buffer/EDTA group) (Figure 4C). Among different divalent metal ions, Mg^2+^ performed the best. Mg^2+^, Ca^2+^, Sr^2+^, and Ba^2+^ belong to the second group elements; Mn^2+^, Zn^2+^, Fe^2+^, Cu^2+^, and Co^2+^ are the fourth‐period elements. The order of DNAzyme activity promotion effect was Mg^2+^ > Mn^2+^ > Sr^2+^ > Ba^2+^ > Ca^2+^ > Zn^2+^, which is consistent with previously report (Santoro & Joyce, 1998). Finally, Mg^2+^ was selected as the divalent metal for the biosensor.

### 3.4 Sensitivity detection of DVc1

**FIGURE 5:**
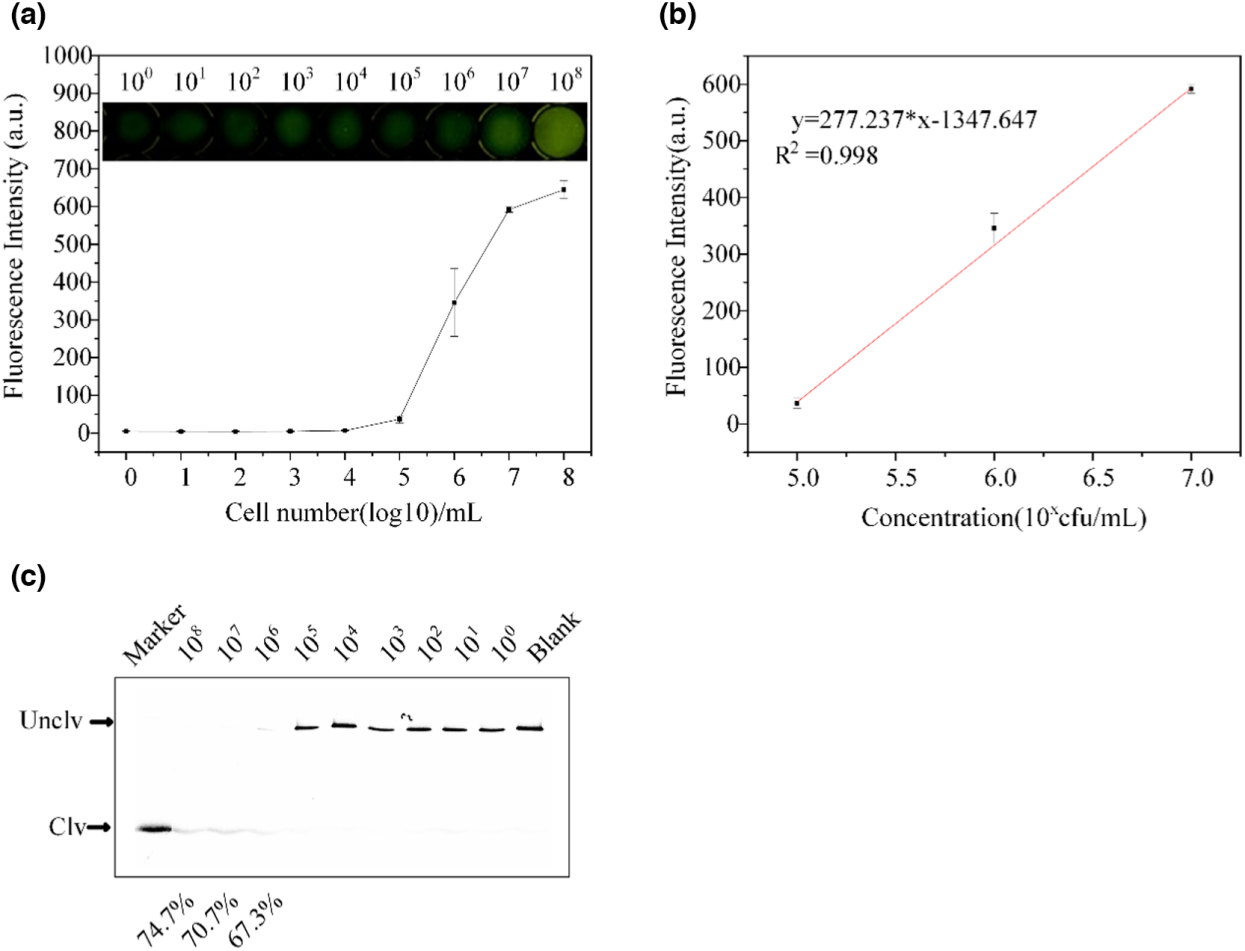
Sensitivity detection of DVc1. (a) Fluorescence values at different concentrations of Vc (Blank: selection buffer without Vc). (b) Analytical calibration curve of fluorescence values at 5.5 × 10^5^, 5.5 × 10^6^, and 5.5 × 10^7^ CFU/mL of Vc. (c) Gel cleavage assay at different concentrations of Vc (Blank: selection buffer without Vc). The dot means: mean ± SD in the legend.

The fluorescence values after 2 h of biosensor assay are shown in Figure 5a. The initial culture medium had 5.5 × 10^8^ CFU/mL of Vc and the colony‐forming units are mentioned in section 2.1. The fluorescence values generated by DVc1 cleavage activity gradually increased with the increased concentration of Vc from 5.5 to 5.5 × 10^8^ CFU/mL after gradient dilution. The corresponding analytical calibration curve (y = 277.61x‐1340.925, R^2^ = 0.996) for cleavage was plotted linearly (Figure 5b), yielding a detection limit of 7.2 × 10^3^ CFU/mL, where LOD = (K*Sb/m) × 5.5 × 10^5^ = (3 × 1.247/277.611) × 5.5 × 10^5^ = 7.2 × 10^3^ CFU/mL; K is a coefficient determined at a certain confidence level (taken as 3), Sb is the blank standard deviation (1.247), and m is the slope of the analytical calibration curve in the concentration range of 10^5^ to 10^7^. The background concentration was 5.5 × 10^5^ CFU/mL. The gel assays are shown in Figure 5C. Also, the amount of cleaved DNAzyme fragments decreased with the decrease in Vc concentration; no degradation was detected up to 5.5 × 10^6^ CFU/mL of Vc.

### 3.5 Properties and molecular weight of the target

**FIGURE 6:**
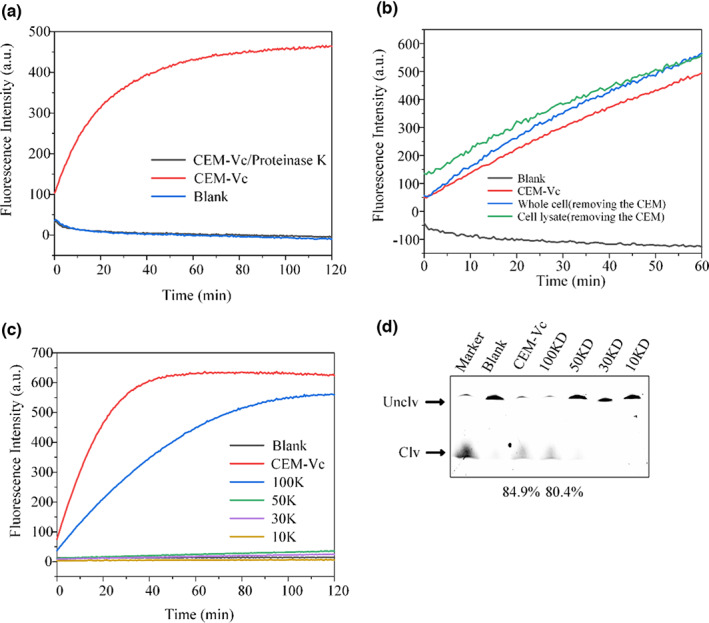
Properties and molecular weight of the target. (a) Proteinase K‐treated CEM‐Vc showed no signal with DVc1. (b) Detection of the whole cell, cell lysate and CEM. Molecular weight assessment of target protein by (c) fluorescence and (d) gel assays.

CEM‐Vc spiked with proteinase K did not produce fluorescence, suggesting that the target of DVc1 is a protein (Figure. 6a). Also, both the whole cell and cell lysate samples showed elevated fluorescence (Figure 6b). These results suggested that the target protein was existing in the CEM and cell membrane surface. The cell lysate did not increase the reaction time, so we speculate that the DVc1 remains unaffected by the cellular RNase. The results of the molecular weight screening experiment showed that the filtrates of <50 kDa filter did not induce the cleavage reaction (Figure 6c); only the filtrate of 100 kDa filter produced the fluorescence signal. The same inference was made from the dPAGE assay (Figure 6d).

### 3.6 Biosensor board design

**FIGURE 7:**
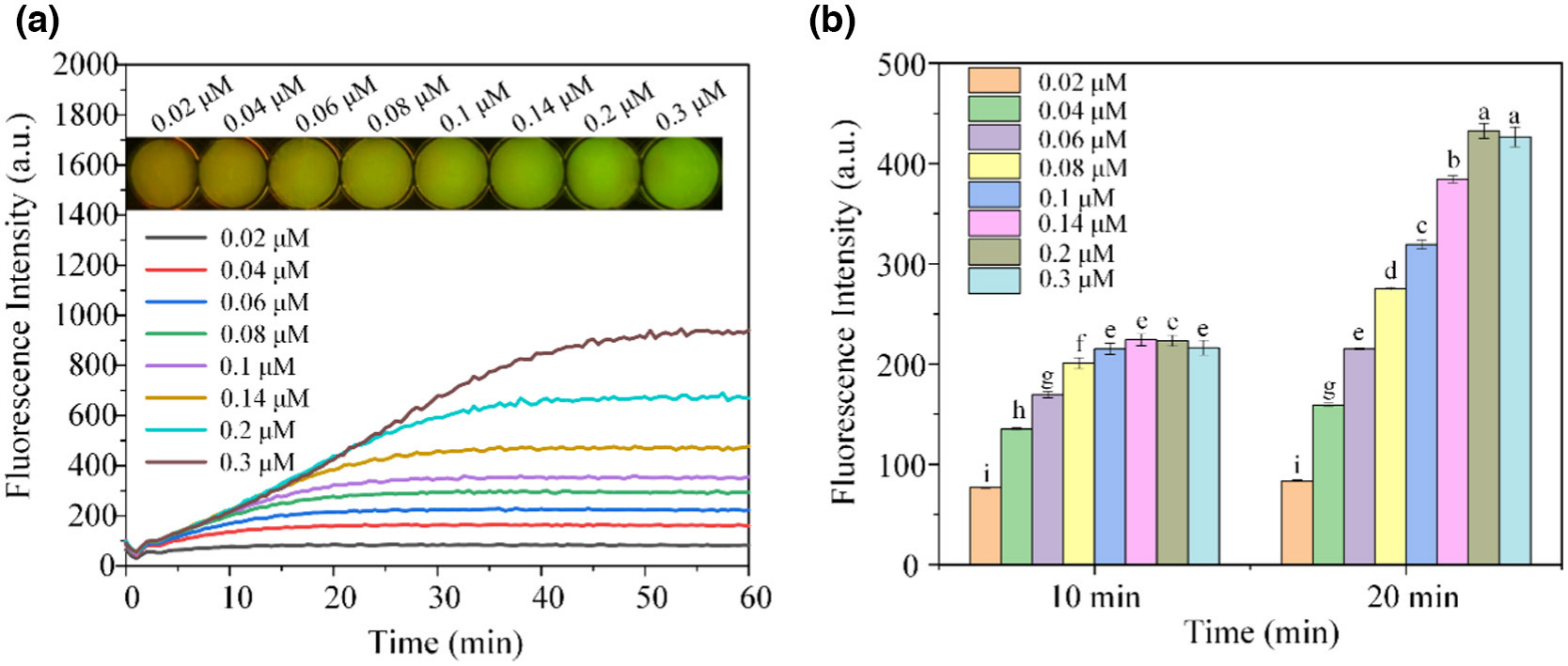
Biosensor board design. (a) Increasing fluorescence signal of DVc1‐S at different concentrations of Vc within 1 h of sample addition. The corresponding pictures of the fluorescence signal are shown at the top. (b) Significant differences in the fluorescence intensity was significant difference with each concentration at 10 min and 20 min [a‐i were considered to be statistically significant (*p* < .05), and the same letters were considered to be not significant (*p* > .05)]. The bar means: mean ± SD in the legend.

The results of the sensor board design are shown in Figure 7. We found that the fluorescence signal was clear even at the lowest concentration of 0.2 μM at 20 min (Figure 7A). Accordingly, 0.2 μM was chosen as the optimal probe concentration and the best reaction time was 20 min. This reduced the need for DVc1‐S (lower cost) and shortened the reaction time. Data in Figure 7B shows that 0.2 μM at 20 min produced the highest fluorescence intensity. Our results indicated that there was significant difference of the fluorescence intensity between 0.2 μM to other concentrations at 20 min.

### 3.7 Detection based on sensor board

**FIGURE 8:**
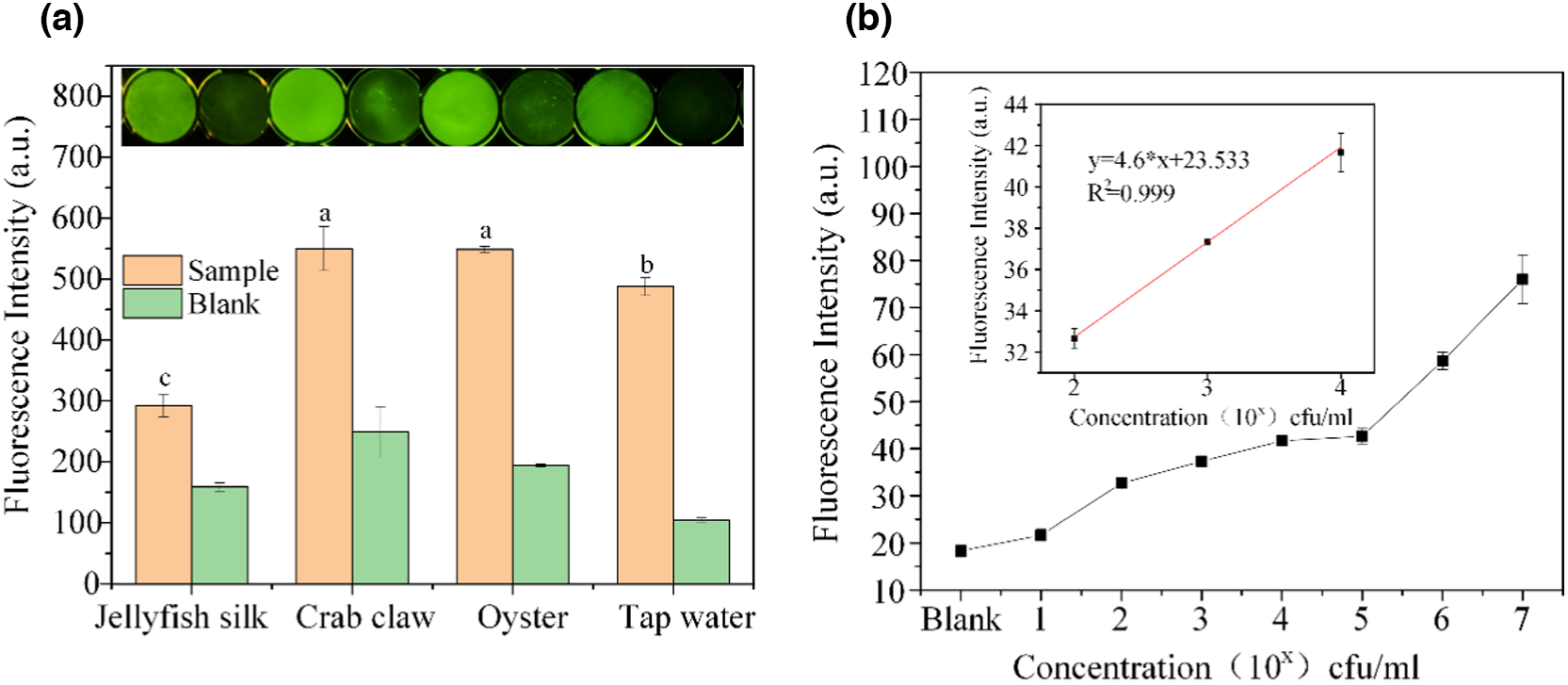
(a) Fluorescence intensities and corresponding photographs of the sensor for the tested samples: Jellyfish silk, crab claw, oyster, and tap water [a‐c were considered to be statistically significant (*p* < .05), and the same letters were considered to be not significant (*p* > .05)]. (b) Fluorescence intensities of gradually diluted oyster samples. Analytical calibration curve of fluorescence values at 1.57 × 10^2^, 1.57 × 10^3^, and 1.57 × 10^4^ CFU/mL of Vc is insert. The bar and the dot mean: mean ± SD in the legend.

The fluorescence and corresponding photograph of aquatic products on the sensor are shown in Figure 8A. Among the experimental groups, sea crab pincers and oysters exhibited the strongest signal, while the control group did not show fluorescence. Oysters were chosen for the subsequent detection as described in section 2.10. Based on different sample dilutions, the detection limit of the sensor for raw choked oysters was calculated as 1.28× 10^2^ CFU/mL (Figure 8B), where LOD = (K*Sb/m) × 1.57 × 10^2^ = (3 × 1.25/4.6) × 1.57 × 10^2^ = 1.28 × 10^2^ CFU/mL.

## 4 DISCUSSION

In 2011, Ali et al. devised a new method for detecting specific bacteria using unpurified CEM. A fluorescent‐labeled DNAzyme, screened from a random sequence DNA library, was used to build a simple mix‐and‐read bacterial assay. More importantly, this method can detect individual living cells and bypass the tedious and time‐consuming probe isolation and subsequent analysis process. The first target bacterial active DNAzyme was RFD‐EC1 (Ali et al., 2011). In this study, the DNAzyme was screened by magnetic bead method using both positive and native screening against CEM‐Vc and CEM of seven other bacteria. This significantly improved the specificity of our DNAzyme (DVc1), which was selected based on a series of screening, sequencing, cleavage activity, and specificity comparison assays.

The ability to grow under nutrient conditions and exchange substances with the environment are unique properties of living cells. Microbes leave behind a mixture of small or large molecules as CEM. Purifying and identifying suitable targets from CEM for biosensor development is too laborious, expensive, and difficult. Recent studies have directly used bacterial CEM as multiple targets for the screening of DNAzyme. CEM may contain potential biomarkers such as proteins, nucleic acids, lipids, and polysaccharides. Studies have shown that proteins are the major targets of DNAzyme in CEM (Ali, Slepenkin, et al., 2019; Ali, Wolfe, et al., 2019; Shen et al., 2016). Accordingly, we performed protein degradation of CEM‐Vc and found that it failed to activate DNAzyme, indicating that the target of DVc1 is indeed a protein. Furthermore, the filter screening assay suggested that it could a protein of >50 kDa.

Compared with other Vc detection methods, which are limited by long detection time, expensive equipment, cumbersome operation, and/or the need for trained personnel, our DNAzyme sensor can perform onsite detection of Vc in a convenient, quick and simple manner.

## 5 CONCLUSION

In summary, the DNAzyme DVc1 was successfully screened in vitro and a simple DNAzyme‐based sensor was designed for the rapid detection of Vc. The sensor has good sensitivity and specificity at pH 8 and a DVc1 concentration of 200 nM. The sensor has a low limit of detection at 7.2 × 10^3^ CFU/mL of Vc. 1.28 × 10^2^ CFU/mL of Vc were successfully detected in raw ready‐to‐eat oysters within 20 min. Our sensor can help the seafood industry with the timely detection of Vc contamination.

## FUNDING INFORMATION

This study was supported by the Jiangsu Agricultural Science and Technology Innovation Fund (CX(22)3079); The Priority Academic Program Development of Jiangsu Higher Education Institutions (PAPD).

## CONFLICT OF INTEREST STATEMENT

The authors declare no conflict of interest.

## Data Availability

The datasets generated during and/or analyzed during the current study are available from the corresponding author upon reasonable request.
